# Synthesis, Characterization, and Application of Poly(4,4'-Cyclohexylidene Bisphenol Oxalate) for Solid-Phase Extraction of DNA

**DOI:** 10.1155/2019/7064073

**Published:** 2019-02-05

**Authors:** Aisha Nawaf Al balawi, Nor Azah Yusof, Sazlinda Kamaruzaman, Faruq Mohammad, Helmi Wasoh, Khulood Fahad Al Abbosh, Hamad A. Al-Lohedan

**Affiliations:** ^1^Department of Chemistry, Faculty of Science, Universiti Putra Malaysia, 43400 UPM Serdang, Selangor, Malaysia; ^2^Haql College, University of Tabuk, Tabuk 71491, Saudi Arabia; ^3^Institute of Advanced Technology, Universiti Putra Malaysia, 43400 UPM Serdang, Selangor, Malaysia; ^4^Surfactants Research Chair, Department of Chemistry, College of Science, King Saud University, Riyadh 11451, Saudi Arabia; ^5^Faculty of Biotechnology and Biomolecular Sciences, Universiti Putra Malaysia, 43400 UPM Serdang, Selangor, Malaysia; ^6^Microbiology Program, Department of Biology, Faculty of Science, University of Hail, Saudi Arabia

## Abstract

The present study has synthesized poly(4,4'-cyclohexylidene bisphenol oxalate) by the condensation of oxalyl chloride with 4,4'-cyclohexylidene bisphenol, where its efficacy was tested for the solid-phase extraction of DNA. The synthesized polymer in the form of a white powder was characterized by FTIR, TGA-DTG, SEM, and BET analysis. The study utilized solid-phase application of the resulting polymer to extract DNA. The analysis of results provided the information that the extraction efficiency is a strong dependent of polymer amount and binding buffer type. Among the three types of buffers tested, the GuHCl buffer produced the most satisfactory results in terms of yield and efficiency of extraction. Moreover, the absorbance ratio of A260/A280 in all of the samples varied from 1.682 to 1.491, thereby confirming the capability of poly(4,4'-cyclohexylidene bisphenol oxalate) to elute pure DNA. The results demonstrated an increased DNA binding capacity with respect to increased percentage of the polymer. The study has concluded that poly(bisphenol Z oxalate) can be applied as one of the potential candidates for the high efficiency extraction of DNA by means of a simple, cost-effective, and environmentally friendly approach compared to the other traditional solid-phase methods.

## 1. Introduction

Deoxyribonucleic acid (DNA) isolation process involves extraction and purification of DNA with the combination of various chemical and physical methods obtained from different sources. Such isolated DNA can be used for the investigation of many different biomolecules during the genetic analysis that has implications in the forensic science and biomedical sector for the diagnostic purposes. For example, DNA extracted from the animal or plant cells are used to detect some diseases or improve their metabolic processes. The samples commonly found for the isolation and testing of DNA include the hair, nail, sperm, bone, blood, tissue, saliva, buccal (cheek) swab, epithelial cells, urine, bacteria, plants, and animal tissues [1].

Molecular biology essentially recovers DNA to identify the plants, animals, or individuals (e.g., criminals, victims of accidents, or war victims) for determining their paternity [2, 3]. Previous studies have successfully investigated the detection of DNA in the biological context, where they indicated that the method of choice for DNA extraction is crucial to ensure proper selection and optimization of the yields and quality of the extracted DNA [1]. The studies indicated that the selection of a right method produces good quality DNA within short period of time. For instance, Friedrich Miescher achievedthe first successful DNA isolation from the leucocytes cells during the year 1869 and since then researchers followed the suit to investigate more efficient DNA extraction methods [4]. Briefly, these can be divided into two types, DNA extraction by liquid phase and solid support-based extraction.

DNA extraction by liquid phase was performed by Birnboim and Dolyin (1979). They studied the alkaline extraction method using alkaline lysis, where the normal process involved the extraction of plasmid DNA from bacterial cells. The method requires specific selection of the alkaline denaturation of high molecular weight chromosomal DNA and controlling the pH without the use of a pH meter [5, 6]. Similarly, the phenol-chloroform extraction includes the liquid-liquid extraction method to isolate DNA using the organic solvent combined with flammable and toxic chemicals. However, it is still the most commonly used liquid-based method for DNA extraction that involves the separation of molecular mixtures on the basis of different solubility properties of the individual molecules in two different layers [7]. Moreover, the ethidium bromide-cesium chloride method takes account of DNA isolation in agarose gel and in many cesium chloride gradient protocols. The ethidium bromide solvent used in this approach is a toxic chemical and is also a mutagen, causing eye and skin irritations [8, 9].

Solid support-based extraction method enables rapid and effective means of DNA purification compared to the liquid phase-based methods. The solid-phase process includes DNA absorption depending on the buffer and pH. The process mechanism depends on the three principles such as hydrogen-binding interaction with a hydrophilic matrix under the chaotropic conditions, ionic exchange under aqueous conditions through an anion exchange, and affinity and size exclusion mechanisms. For the solid support in this method, the adsorbents such as glass particles [10], diatomaceous earth [11, 12], silica matrices [13], magnetic beads [14], anion exchange materials [15], and graphite oxide-based materials [16] have been used. The first step in a solid-phase process uses binding buffer that helps to bind DNA to the solid surface by providing the required conditions. These conditions depend on the solid surface and buffer composition, pH, etc., followed by washing with ethanol and buffer. However, further elution is performed by using elution buffer such as the TE (Tris-EDTA) buffer, AE buffer (10 mM Tris-Cl and 0.5 mM EDTA; pH 9.0), or water to free the required nucleic acid molecules from the sample. The solid-phase extraction process is performed on the target nucleic acid under various solution conditions to free nucleic acid under the same elution conditions [17].

Poly(bisphenol Z oxalate) can be an alternative and novel approach for the rapid and selective extraction of DNA. This involves the agitation of DNA to be selectively transported from a sample through an active side of poly(bisphenol Z oxalate). The different kinds of polymers containing the oxalyl groups applied for the elimination/extraction of small molecules are generally prepared by the poly-condensation of oxalyl chloride, oxalic esters with diols/oxalic acid, and stepwise polymerization of polyfunctional monomers by different condensation reactions [18]. The polyoxalates contain two adjacent carbonyl groups in the constitutionally repeat units and these polymers have a relatively high average molecular weight. Therefore, they show acceptable mechanical features, which make them suitable for the practical use [18–20]. In the present study, poly(4,4'-cyclohexylidene bisphenol oxalate) polymer was synthesized with single phase organic solvent condensation polymerization method [21]. The characterization of the polymer was performed by different instrumental techniques and DNA adsorption characteristics by means of applying the changes in polymer weight and buffer mediums were further studied. Finally, the testing of isolated DNA and the purity were confirmed by measuring the optical intensity in elusion at two wavelengths, i.e., 260 nm and 280 nm.

The present study complements a series of research and proposes additional latest requirements in the sample preparation trends including miniaturization, simplification, and environmentally friendly alternatives. The structure of DNA leads to the development of a method to determine DNA, which allows the utilization of poly(bisphenol Z oxalate) that is adsorbed. This study makes use of one type of bisphenol Z only, while the other type of bisphenol compounds includes a range of analogues such as bisphenol A, bisphenol B, bisphenol F, bisphenol S, bisphenol AF, bisphenol P, bisphenol C, bisphenol AP, bisphenol E, and two hydroxy phenyl functionality containing compounds [19]. The study aims to test the efficiency of the polymer as a solid-phase adsorption material by considering the mechanical features and chemical functionality offered by the poly(bisphenol Z oxalate) compound.

## 2. Material and Methods

### 2.1. Materials

The materials used in this study were of analytical grade and applied without any further purification. The chemicals purchased from Sigma Aldrich were oxalyl chloride, 4,4'-cyclohexylidene bisphenol, guanidine hydrochloride (GuHCl), pyridine, methanol, ethanol (EtOH), dehydrated chloroform, and tetrahydrofuran (THF). The ssDNA (single-stranded DNA) solution was obtained from Sigma company (D7290) and was applied as the ssDNA specimen. Sonication shears the large molecular weight DNA to produce fragments in a size range of 587 to 831 base pairs and with concentrated solution (9-12 mg/mL DNA).

### 2.2. Preparation of Poly(4,4'-Cyclohexylidene Bisphenol Oxalate)

A solution of oxalyl chloride (0.04 mol) in dried THF (20 mL) was added drop-wise to a mixture of 4,4'-cyclohexylidene bisphenol (0.04 mol) and pyridine (0.12 mol) in dried THF (40 mL) maintained at 0-5°C, using an ice bath. The addition was followed by the stirring of reaction mixture for 1 hour at 0-5°C temperature. The ice bath was removed after the period and the mixture was allowed to stand for another 24 hours. After the period, the reaction mixture was diluted with chloroform (100 mL) and further transferred to a separating funnel containing distilled water (100 mL). In the separating funnel, the organic layer is washed starting with 2 x 100 mL distilled water, 1 x 100 mL 5 % (v/v) HCl, and again with 4 x 100 mL distilled water. Therefore, obtained organic portion was stirred with anhydrous magnesium sulfate to dry the chloroform layer. The chloroform solution was concentrated to about 40 mL (using rotatory evaporator). Finally, the drop-wise addition of methanol to the organic portion resulted in the precipitation of product, which was collected by filtration and dried at 60°C under vacuum to obtain the white powder (6.303 g) of poly(4,4'-cyclohexylidene bisphenol oxalate) with 48% yield.

### 2.3. DNA Extraction Studies

The solid-phase DNA purification took place in three stages:

- Adsorption of DNA to the solid matrices
- Rinsing the excess salts and proteins
- Desorption of DNA from the solid matrices

The process also includes preparation of three binding buffers; 2 M GuHCl in 96% EtOH [22], 2 M NaCl solution, and phosphate-buffered saline (PBS-5 M GuHCl in 30% propanol). These three buffers were used to measure the binding capacity of poly (bisphenol Z oxalate) for DNA analysis. For the extraction, a 200 *µ*L saturated DNA solution (20 *µ*L DNA solution and 180 *µ*L H_2_O) was mixed with 300 *µ*L binding buffer in an Eppendorf tube that already contains different weights of polymer (0.02 g, 0.10 g, and 0.20 g). This mixture is allowed to incubate for another 10 minutes and following the period; the solution was siphoned out using a pipette and washed with 70% EtOH to remove the leftover salts from the surface of the polymer. Subsequently, 500 *µ*L of elution buffer AE (composition: 10 mM Tris-HCl, 0.5 mM EDTA, pH 9.0) was added to the tubes and incubated for another 5 minutes, followed by separation of elution from the polymer. The assessment of the quality and quantity of DNA in the elution buffer was performed in terms of efficiency and purity of the extraction, respectively.

### 2.4. Qualitative and Quantitative Analysis of the Extracted DNA

A nanophotometer device was applied to measure the absorbance ratio between 260 nm and 280 nm that should range from 1.8 to 2.0 for the high purity DNA for the qualitative and quantitative analysis [22–24]. The total yield of DNA purification was measured by a final elution of the solution's volume multiplied by DNA concentration (ng/*μ*L). However, the extraction efficiency was calculated by dividing the total DNA yield by input volume or total DNA amount (ng/input DNA volume, *μ*L). All of the experiments were carried out in triplicate so that the data could be replicated.

### 2.5. Instrumental Analysis

To understand the extent of binding, the fourier transform-infrared (FTIR) spectroscopic analysis was performed and for that, the PerkinElmer Spectrum 100 FTIR instrument was used. For the analysis, a transparent pellet was prepared by mixing and grinding of polymer with KBr, and the data was recorved in the wavelength range of 4000-400 cm^−1^. For the ^1^H and ^13^C NMR spectroscopic analysis, the Jeol Module 500ss instrument was utilized. Scanning electronic microscopy (SEM) analysis was performed on a Module NOVA NANOSEM 230 – FE 1™ instrument. The surface area and pore size distribution for the polymer sample were analyzed by the BET (Brunauer-Emmett-Teller) and BJH (Barrett-Joyner-Halenda) techniques, where a Micromeritics-3ΔFlex-500583523 Module was applied. The thermogravimetric analysis (TGA) and differential thermal gravimetry (DTG) were investigated under dry nitrogen to detect changes in polymer weight loss and phase changes with respect to the temperature using Mettler Toledo instrument. The ultraviolet-visible (UV-Vis) spectroscopic analysis of pure and DNA bound polymer samples was carried on a NANODROP 2000 spectrophotometer from Thermo Scientific. UV-Vis was measured through Nanodrop 2000 spectrophotometer.

## 3. Results and Discussion

**Scheme 1:**
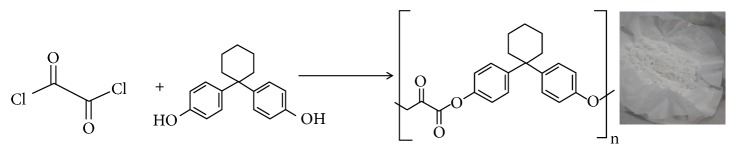
Schematic representation for the condensation reaction of oxalyl chloride and 4,4'-cyclohexylidene bisphenol to form poly(4,4'-cyclohexylidene bisphenol oxalate).

For the synthesis of poly(4,4'-cyclohexylidene bisphenol oxalate) polymer, we followed the procedure shown schematically in Scheme 1, where the pyridine-catalyzed nucleophilic displacement of chloride of oxalyl chloride by the alcoholic group of bisphenol Z group diol was followed. The attainment of a relatively high polymer yield may be due to the high reactivity of oxalyl chloride group. The resulting polymer was obtained in the form of powder and found insoluble in many common organic solvents like THF, diethyl ether, acetone, and methanol, but soluble in chloroform.

**Figure 1:**
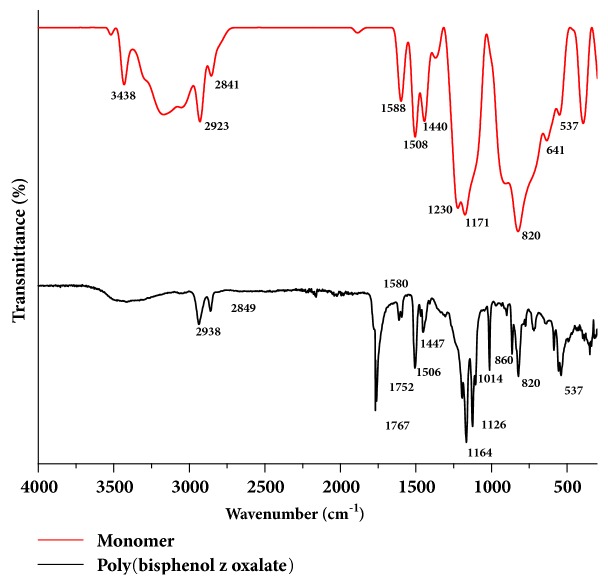
FTIR spectral comparison of bisphenol with that of poly(4,4'-cyclohexylidene bisphenol oxalate).

The step-wise analysis for the formation of polymer was confirmed by FTIR spectroscopy where the characteristic absorption bands are illustrated in Figure 1. From the figure, the formation of the polymer may be primarily confirmed by comparing the characteristic bands for the ester group and the C-O-C bond. The FTIR spectrum of the polymer exhibited two robust absorption bands for the stretching vibrations of the two carbonyl groups (C=O) of oxalate at 1760 cm^−1^ and 1752 cm^−1^ and a C-O-C stretching frequency in the finger print region at around 1163 cm^−1^. These frequencies are typical for the oxalate group and are in a good agreement with the literature studies [25]. These three absorption bands of oxalate have not appeared in the bisphenol Z monomer, indicating that the oxalate is getting attached successfully to the polymer backbone. In addition, the presence of –OH stretching band around 3438 cm^−1^ in the IR spectrum of the monomer and the absence of the same band in the polymer confirms the occurrence of polymerization.

**Figure 2:**
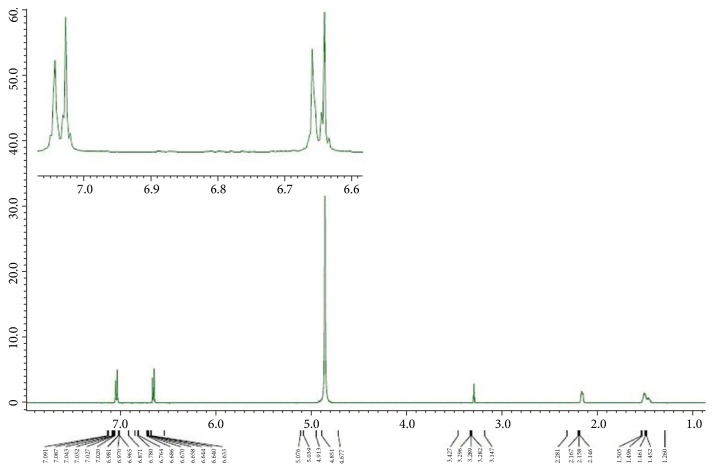
The ^1^H NMR spectrum of poly(4,4'-cyclohexylidene bisphenol oxalate).

**Figure 3:**
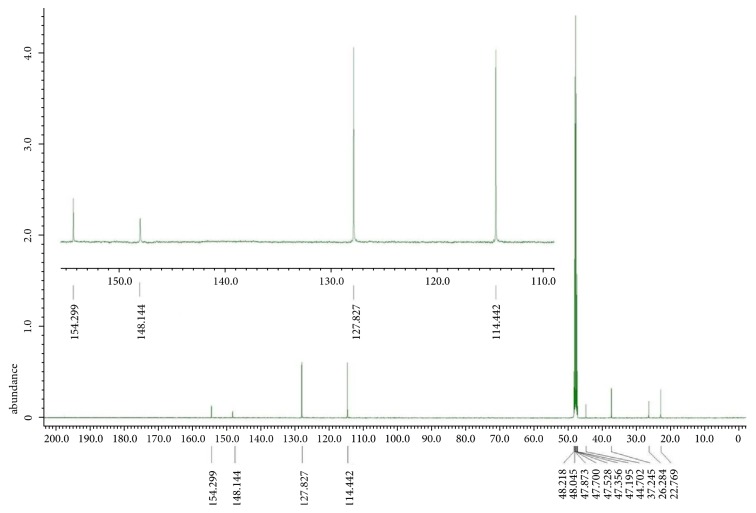
The ^13^C NMR spectrum of poly(4,4'-cyclohexylidene bisphenol oxalate).

For confirming the structure and associated bonding of formed polymer, the product was analyzed by NMR spectroscopy and the results are shown in Figures 2 and 3. In the ^1^H NMR spectrum of Figure 2, we observed a pattern of peaks for the polymer moiety having no peaks for the oxalate proton groups, while the aromatic protons of polymer unit were shown in the range of 6.6–7.1 ppm and the proton of cyclohexane rings at 1.46 ppm. Similarly, the ^13^C NMR analysis of polymer shown in Figure 3 shows three different units of carbons, the oxalate unit, aromatic benzene ring unit, and cyclohexane ring unit. In Figure 3, the carbons of oxalate groups in the polymer and the carbons of aromatic rings attached to the oxygen of the oxalate function appeared at 154 ppm and 148 ppm respectively. The carbons of aromatic rings were observed at 114 ppm and 128 ppm. The peaks of all the carbon atoms of the cyclohexane rings appeared around 23-37 ppm. The observation of  ^13^C  NMR peak of carbon of aromatic ring in a polymer attached to the oxygen of oxalate group is a strong indicative for the formation of the expected structure of polymer. The free oxalyl chloride appeared slightly downfield at 159 ppm but the peak of carbonyl carbon of oxalyl group attachment of bisphenol Z led to an upfield shift at 154 ppm. This analysis is conformed with the data reported in the literature studies [26, 27]. Furthermore, the advantage of ^13^C NMR spectrum of poly(4,4'-cyclohexylidene bisphenol oxalate) is also worth mentioning. The appearance of a singlet peak for the carbon atoms of oxalate unit in the poly(4,4'-cyclohexylidene bisphenol oxalate) due to that these units are present in the polymer backbone along with the polymer chain with alternatives to the bisphenol Z and oxalate groups.

**Figure 4:**
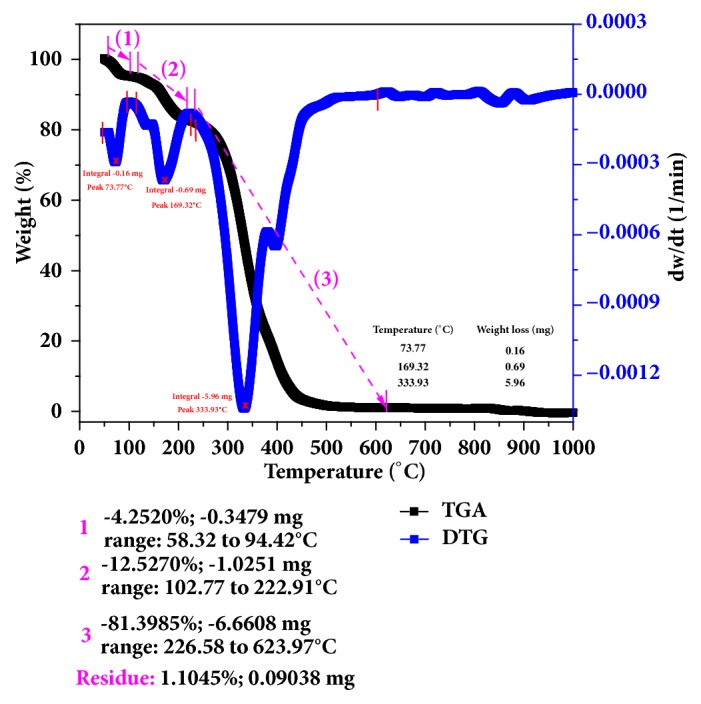
TGA and DTG analyses for the synthesized polymer.

The thermal properties of synthesized polymer were examined by TGA and DTG as shown in Figure 4. The figure showed that the decomposition of the polymer sample occurred in three mass loss steps. The first thermal decomposition step of polymer was observed around 58.32-94.42°C, with sample mass loss value of 4.252%. The second thermal decomposition event occurred in the range of 102.77-222.91°C, with a mass loss value of 12.527%. The last decomposition event was observed in the range of 226.58-623.97°C (327.72°C) with sample mass loss value of 81.3985%. The residual mass of sample remained was 0.09 mg corresponding to 1.1% of the initial mass of polymer sample. The DTG curve often improves the evaluation of the step in a TGA curve and makes it easier to determine the limits of the TGA steps (shown as minima between the peaks in the DTG curve) in Figure 4. The beginning of the pyrolysis reaction can be clearly identified in the DTG curve as it is invisible in the TGA curve. The first integral peak in DTG curve is observed at 73.77°C with a mass loss of 0.16 mg, while the second integral peak is at 169.32°C with mass loss of 0.69 mg. The end of the pyrolysis reaction of polymer occurred at 333.93°C which corresponds to the mass loss of 5.96 mg. The TGA and DTG analysis of the data presented in the figure indicates that the polymer is thermally stable up to 330°C as there is not much significant loss in the total weight and thereby confirming the safe use of the polymer up to this temperature.

**Figure 5:**
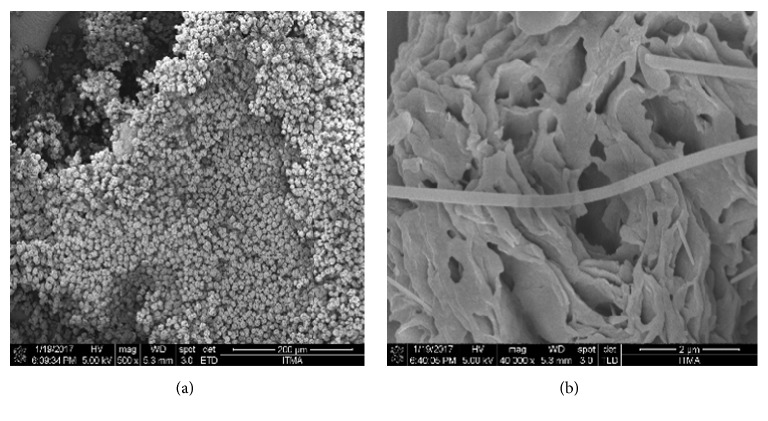
SEM images of poly(4,4'-cyclohexylidene bisphenol oxalate) at a scale of (a) 200 *µ*m and (b) 2 *µ*m.

The surface morphology of the polymer was analyzed with SEM at two different magnifications as shown in Figures 5(a) and 5(b). The figure clearly shows that the polymer is formed as resin (Figure 5(a)) and the channel like structure is observed on zooming (Figure 5(b)). The microscopic image shown in Figure 5(b) indicated that the interior structure of the polymer maintains channels and large gaps, which are randomly distributed in the structure of polymer. The polymer is a white powder having spherical shaped resins (Figure 5(a)) and channels formed on its surface in the diameter range 52-110 nm (Figure 5(b)) at its surface. When tested for the adsorption related applications, the porous channels are used to increase DNA adsorption capacity by means of providing the space for the localization of DNA molecules. The results also indicated that the particles of poly(4,4'-cyclohexylidene bisphenol oxalate) have relatively homogenous size (Figure 5(a)). Moreover, the diameter of polymer particles can be easily controlled by the volume and the tip size of the Eppendorf syringe.

**Figure 6:**
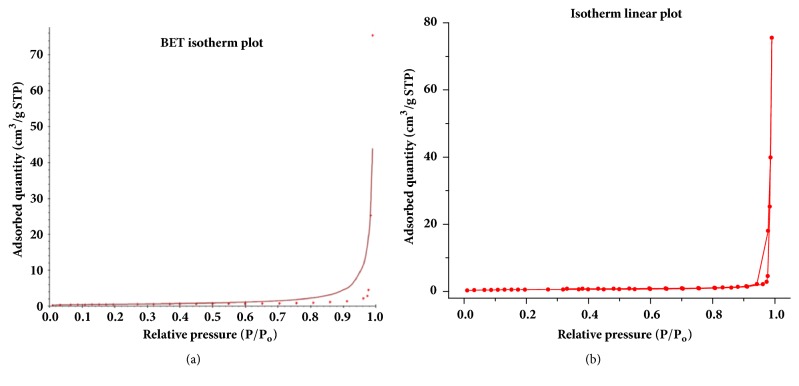
(a) BET isotherm plot and (b) BET isotherm linear plot for polymer.

**Figure 7:**
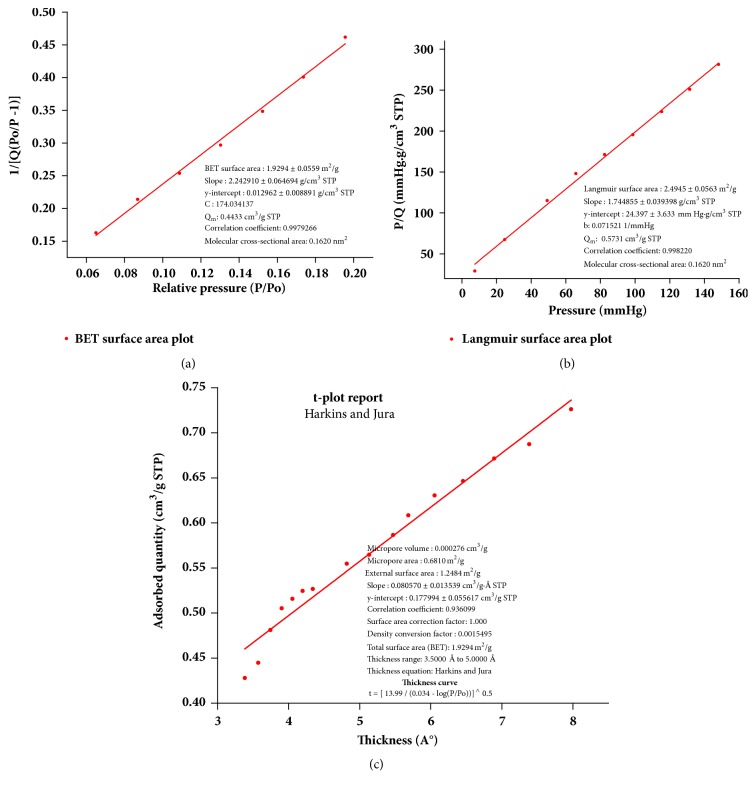
(a) BET isotherm, (b) Langmuir surface area plot, and (c) t-plot from type 3 isotherm for the polymer.

The surface area of poly(4,4'-cyclohexylidene bisphenol oxalate) was measured by BET analysis, the results are shown in Figures 6 and 7, and Table 1. Here, N_2_ gas was applied as adsorptive and the bath was maintained at 77.332 K temperature for conducting the analysis. From Figures 6(a) and 6(b), the appearance of a linear isotherm curves confirms to the IUPAC type III adsorption isotherm with a relative pressure range 0.0 < P/P_0_ < 1 indicating that the polymer has a mixed macrospores and mesoporous structures. The curve also indicated that nitrogen gas uptake increased slowly with respect to an increase in the applied relative pressure, thereby indicating that the adsorption mechanism is a multilayered one [28]. Table 1 shows the values of surface area and pore volume from the BET analysis, where the results indicated weak interaction between the polymer and the gas particles. Table 1 also shows that N_2_-mediated BET surface area of the polymer is 1.9294 m^2^/g at a relative pressure P/P_0_ of 0.27, total Langumir surface area is 2.4945 m^2^/g, and average pore diameter is 2426.3042 Å, (242.63 nm). Thus, the majority of the pores were classified in the range of macroporous with an average pore diameter of 242.63 nm related to IUPAC classification on pore dimensions. The three kinds of pore dimensions of adsorbents included the micropore (d < 2 nm), mesopore (d = 2-50 nm), and the macropore (d > 50 nm).

The adsorption/desorption pore size distribution results for the synthesized polymer are shown in Tables 2 and 3. The pore size distribution analysis in the tables was observed in the range of average pore radius 129.7 Å-685.1 Å for BJH adsorption and in the range of average pore radius for desorption 120.8 Å-772.2 Å. These results confirm the presence of the mesoporous and macrospores in the absorbent particles. Li* et al.* showed that the hybrid core materials with mixed (fumed silica), polyester chopped strand fibers, hollow glass microsphere, titanium dioxide, and carbon black powders had an average pore size of 19.0-181.1 nm [23, 29].

### 3.1. Extraction Efficiency and Purity of DNA

**Figure 8:**
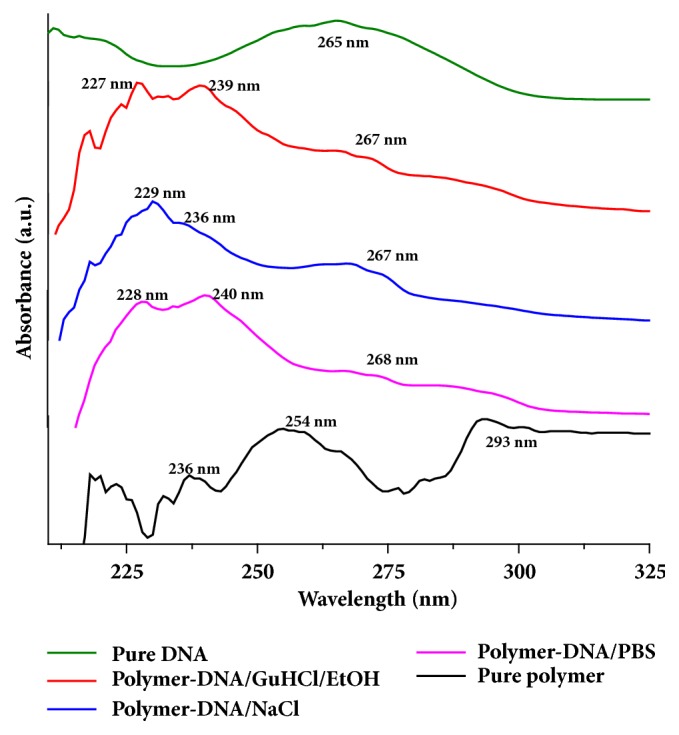
Comparison of the UV-Vis absorption graph in the range of 260-280 nm for the qualitative and quantitative pure DNA, DNA with binding buffer solutions (GuHCl/96% EtOH, PBS, and NaCl), and pure polymer solution.

The UV-Vis absorption spectroscopy was also employed to test DNA adsorption/binding capability of the polymer synthesized. The spectroscopic results have been presented in Figure 8. The figure shows that the peak observed around 265 nm for the pure DNA sample was persisting in the other polymer-DNA samples too (around 267 nm and 268 nm), when tested in the presence of three different binding buffers[30, 31]. In addition, the absorption peaks observed for the pure polymer sample around 236 nm and 254 nm were also observed in the polymer-DNA bound samples at peak positions of 228 nm and 236 nm, respectively. These results confirmed the effective binding of DNA to poly(4,4'-cyclohexylidene bisphenol oxalate) compound synthesized in the present study[32].

**Figure 9:**
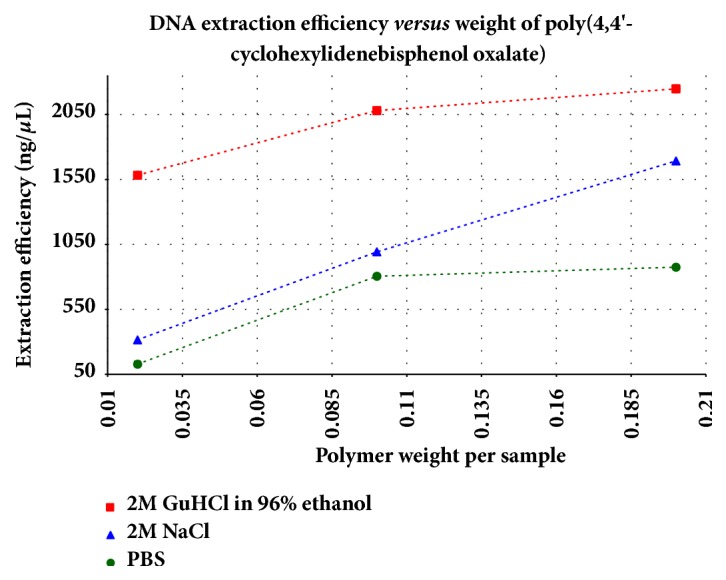
Comparison of weight of polymer (0.02 – 0.2 g) and binding buffer solutions (GuHCl/96% EtOH, PBS, and NaCl) versus DNA extraction efficiency.

Similarly, the changes in the adsorption capacity of poly(4,4'-cyclohexylidene bisphenol oxalate) against the applied parameters (polymer weight and buffers) are shown in Figure 9 and the values are presented in Table 4. The results indicated that the extraction efficiency increases with the mass of the polymer where the efficiency followed the reactivity order of 0.2 g > 0.1 g > 0.02 g. The observation of such reactivity order for the poly(4,4'-cyclohexylidene bisphenol oxalate) can be attributed to the increase in the number of porous channels and associated adsorbent surface area of the mass of polymer adsorbent. In addition, the extraction efficiency of the eluted DNA was also found to depend on the type of binding buffer as the efficiency order for different types of binding buffers (i.e., GuHCl/ EtOH > NaCl > PBS). The increase in the efficiency of GuHCl/EtOH buffer can be due to disable hydrogen bonding of water and the weakened hydrophobic effect that reduces the stability of proteins [33]. The highest amount of eluted DNA was observed to be 16.2% for the 0.2 g of polymer in GuHCl/EtOH buffer; while, the least amount absorbed was 0.93% for the 0.02 g of polymer in PBS. The absorbance ratio of A260/A280 for the 0.2 g polymer during the DNA purification from the three buffers GuHCl/EtOH, NaCl, and PBS was calculated to be 1.601, 1.682, and 1.491, respectively. The values of absorbance ratios were below 1.8 indicating the presence of some impurities in the GuHCl/EtOH, NaCl and PBS buffers that contained DNA solution [23]. Moreover, the highest extraction capacity of 2247.5 ng/*µ*L (1.6, 16.2%) for GuHCl in 96% EtOH was observed by using the polymer synthesized in the present study, while the highest purity of DNA (A260/A280), 1.682 (1692.5 ng/*µ*L, 12.25%), was observed for NaCl buffer.

**Figure 10:**
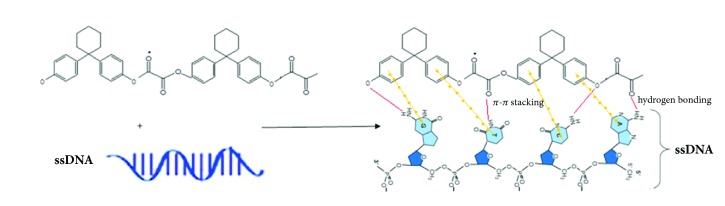
Schematic representation of the mechanism of adsorption and desorption process between polymer and DNA.

The polymer structure consists of ester group, aromatic ring, and oxalate group where the availability of these functional groups suggests several mechanisms for the interpretation of DNA binding mechanism onto the polymer like, hydrogen bonding, electrostatic forces, *π*- *π* stacking, etc., since the electrostatic interaction between DNA and aromatic ring available in the polymer is considered to be the main driving force to have effective *π*- *π* stacking force that promotes for the adsorption. Also, the generation of hydrogen bonding between the polymer's oxygen group and the hydrogen's amine group in the DNA molecule promotes for the effective adsorption (Figure 10). The binding buffers such as GuHCl/EtOH, PBS, and NaCl were added to the solution to enhance the adsorption between DNA and the polymeric surface, where the binding buffers (salt + alcohol, salt or alcohol) like GnHCl which have chaotropic properties play two important roles in the nucleic acid extraction. First, they destabilize hydrogen bonds in nonpolar media; i.e., the hydrogen bonding becomes stronger so GuHCl/EtOH which increases the chemical polarity of the solvent can also destabilize hydrogen bonding through decreasing the water activity and become insufficient water molecules to effectively solvate the ions. Second, they disrupt the association of nucleic acids with water. Ethanol is added for two reasons: (1) to influence and enhance the binding of nucleic acids to the polymer and (2) to correct the concentration which allows for the washing of salts from the membrane [34, 35].

## 4. Conclusion

The present study has prepared a polymer containing oxalate functional group, poly(4,4'-cyclohexylidene bisphenol oxalate), which is capable of extracting DNA molecules from the solution using three types of binding buffers. The physical characterization using the FTIR, NMR, SEM, and thermal analysis provided bonding, shape, and stability related properties, respectively, of the synthesized polymer. The extraction capacity of the polymer using different binding buffers of the sample investigated was found to be within the range of 2247.5-128.5 ng/*µ*L. The observation of such results indicated the successful selection of DNA quality. Furthermore, DNA extraction efficiency got increased with an increase in the polymer mass, where the maximum performance was observed for 0.2 g of polymer with the use of GnHCl/EtOH buffer. The analysis of results indicated that the developed approach was fast, quite simple, and cost-effective; therefore, it could be applied for the solid-phase extraction of DNA in different samples.

## Figures and Tables

**Table 1:** The values of surface area and pore volume for poly(4,4'-cyclohexylidene bisphenol oxalate).

Relative pressure P/P_0_	Surface area	BET surface area	Single point adsorption total pore volume of pores less than 959.130 Å radius	Single point desorption total pore volume of pores less than 959.130 Å radius	The pore size (adsorption and desorption average pore diameter) by (4V/A by BET)
0.27	1.7628 m^2^/g	1.9294 m^2^/g	-	-	-
0.99	-	-	0.117035 cm^3^/g	0.117035 cm^3^/g	-
					2426.3042 Å

**Table 2:** BJH's adsorption-pore distribution report.

Pore radius range (Å)	Average radius (Å)	Incremental pore volume (cm^3^/g)	Cumulative pore volume (cm^3^/g)	Incremental pore area (m^2^/g)	Cumulative pore area (m^2^/g)
950.9 - 585.4	685.1	0.080262	0.080262	2.343	2.343
585.4 - 421.2	476.8	0.033262	0.113523	1.395	3.738
421.2 - 360.5	386.1	0.002693	0.116216	0.139	3.878
360.5 - 249.3	284.9	0.000726	0.116943	0.051	3.929
249.3 - 107.8	129.7	0.000087	0.117030	0.013	3.942

**Table 3:** BJH's desorption-pore distribution report.

Pore radius range (Å)	Average radius (Å)	Incremental pore volume (cm^3^/g)	Cumulative pore volume (cm^3^/g)	Incremental pore area (m^2^/g)	Cumulative pore area (m^2^/g)
950.0 - 681.3	772.2	0.056613	0.056613	1.466	1.466
681.3 - 441.1	511.5	0.034964	0.091577	1.367	2.833
441.1 - 165.7	199.0	0.025328	0.116905	2.545	5.379
165.7 - 103.4	120.8	0.000135	0.117040	0.022	5.401

**Table 4:** Extraction efficiency and total yield of DNA when three different types of binding buffers were tested at three different polymer weights.

Binding buffer	Polymer weight	Concentration (ng/*μ*L)	260/280 wavelength ratio	DNA yield (ng)	Extraction capacity ng/*μ*L	Extraction efficiency (%)
2 M GuHCl/ EtOH	0.02 g	633	1.431	316500	1582.5	11.4%
2 M NaCl	0.02 g	126	1.589	63000	315	2.2%
PBS	0.02 g	51.4	0.613	25700	128.5	0.93%
2 M GuHCl/ EtOH	0.10 g	832	1.499	416000	2080	15.0%
2 M NaCl	0.10 g	397	1.520	198500	992.5	7.1%
PBS	0.10 g	322	1.322	161000	805	5.8%
2 M GuHCl/EtOH	0.20 g	899	1.601	449500	2247.5	16.2%
2 M NaCl	0.20 g	677	1.682	338500	1692.5	12.2%
PBS	0.20 g	350	1.491	175000	875	6.3%

## Data Availability

The data used to support the findings of this study are available from the corresponding author upon request.

## References

[1] Butler J. M. (2011). *Advanced Topics in Forensic DNA typing: Methodology*.

[2] Butler J. M. (2005). *Forensic DNA Typing: Biology, Technology, and Genetics of STR Markers*.

[3] Ohnishi Y., Totoki Y., Toyoda A. (2010). Small RNA class transition from siRNA/piRNA to miRNA during pre-implantation mouse development. *Nucleic Acids Research*.

[4] Dahm R., Avery O., Macleod C. (2010). From Discovering to Understanding. *EMBO Reports*.

[5] Bimboim H. C., Doly J. (1979). A rapid alkaline extraction procedure for screening recombinant plasmid DNA. *Nucleic Acids Research*.

[6] Hudlow W. R., Buoncristiani M. R. (2012). Development of a rapid, 96-well alkaline based differential DNA extraction method for sexual assault evidence. *Forensic Science International: Genetics*.

[7] Barbosa C., Nogueira S., Gadanho M., Chaves S. (2016). DNA extraction: finding the most suitable method. *Molecular Microbial Diagnostic Methods*.

[8] Lahiri D. K., Numberger J. I. (1991). A rapid non-enzymatic method for the preparation of HMW DNA from blood for RFLP studies. *Nucleic Acids Research*.

[9] Hayyan M., Looi C. Y., Hayyan A., Wong W. F., Hashim M. A. (2015). In Vitro and in Vivo toxicity profiling of ammonium-based deep eutectic solvents. *PLoS ONE*.

[10] Dederich D. A., Okwuonu G., Garner T. (2002). Glass bead purification of plasmid template DNA for high throughput sequencing of mammalian genomes. *Nucleic Acids Research*.

[11] Kolenbrander P. E., Parrish K. D., Andersen R. N., Greenberg E. P. (1995). Intergeneric coaggregation of oral Treponema spp. with Fusobacterium spp. and intrageneric coaggregation among Fusobacterium spp.. *Infection and Immunity*.

[12] Zhao F., Koo B., Liu H., Eun Jin C., Shin Y. (2018). A single-tube approach for in vitro diagnostics using diatomaceous earth and optical sensor. *Biosensors and Bioelectronics*.

[13] Esser K.-H., Marx W. H., Lisowsky T. (2006). maxXbond: First regeneration system for DNA binding silica matrices. *Nature Methods*.

[14] Berensmeier S. (2006). Magnetic particles for the separation and purification of nucleic acids. *Applied Microbiology and Biotechnology*.

[15] Birnboim H. C. (1983). A rapid alkaline extraction method for the isolation of plasmid DNA. *Methods in Enzymology*.

[16] Akceoglu G. A., Li O. L., Saito N. (2016). High Efficiency DNA Extraction by Graphite Oxide/Cellulose/Magnetite Composites Under Na+ Free System. *JOM: The Journal of The Minerals, Metals & Materials Society (TMS)*.

[17] Tan S. C., Yiap B. C. (2009). DNA, RNA, and protein extraction: the past and the present. *BioMed Research International*.

[18] Pawlow J. H., Sadow A. D., Sen A. (1997). Palladium(II)-Catalyzed Carbonylation of Alkane Dinitrite Esters to Polyoxalates. *Organometallics*.

[19] Suehiro K., Chatani Y., Tadokoro H. (1975). Structural studies of polyesters. VI. Disordered crystal structure (form II) of poly(*β*-propiolactone). *Polymer Journal*.

[20] Rochester J. R., Bolden A. L. (2015). Bisphenol S and F: A systematic review and comparison of the hormonal activity of bisphenol a substitutes. *Environmental Health Perspectives*.

[21] Sweileh B. A., Al-Hiari Y. M. (2006). Synthesis and thermal properties of polycarbonates based on bisphenol A by single-phase organic solvent polymerization. *Journal of Polymer Research*.

[22] Alexander P. J., Rajanikanth G., Bacon C. D., Bailey C. D. (2007). Recovery of plant DNA using a reciprocating saw and silica-based columns. *Molecular Ecology Resources (Formerly known as Molecular Ecology Notes)*.

[23] Mackey K., Chomczynski P. (1997). Effect of pH and ionic strength on the spectrophotometric assessment of nucleic acid purity. *Biotechniques*.

[24] Lucena-Aguilar G., Sánchez-López A. M., Barberán-Aceituno C., Carrillo-Ávila J. A., López-Guerrero J. A., Aguilar-Quesada R. (2016). DNA Source Selection for Downstream Applications Based on DNA Quality Indicators Analysis. *Biopreservation and Biobanking*.

[25] Simon M. S., Seyferth H. M. (1958). Carbonyl Stretching Frequencies of Some Oxalate Esters. *The Journal of Organic Chemistry*.

[26] Gordon B., Sharma P., Hansen L. (1990). Hydrodegradable copolymers by simple transesterification: PBT-co-polyoxalate. *Polymer Preprints*.

[27] Latha G., Natarajan M., Balaji K., Murugavel S. C. (2014). Synthesis, spectral and thermal characterization of polyester derived from 1,1-bis(4-hydroxyphenyl)cyclohexane. *High Performance Polymers*.

[28] Sun Y., Wada M., Kuroda N., Hirayama K., Nakazawa H., Nakashima K. (2001). Simultaneous determination of phenolic xenoestrogens by solid-phase extraction and high-performance liquid chromatography with fluorescence detection. *Analytical Sciences*.

[29] Li C., Li B., Pan N. (2016). Thermo-physical properties of polyester fiber reinforced fumed silica/hollow glass microsphere composite core and resulted vacuum insulation panel. *Energy and Buildings*.

[30] Chen W., Schuster G. B. (2013). Structural stabilization of DNA-templated nanostructures: crosslinking with 2,5-bis(2-thienyl)pyrrole monomers. *Organic and Biomolecular Chemistry*.

[31] Berti L., Alessandrini A., Facci P. (2005). DNA-templated photoinduced silver deposition. *Journal of the American Chemical Society*.

[32] Wang Y.-Q., Zhang H.-M., Cao J., Tang B.-P. (2014). Binding of a new bisphenol analogue, bisphenol S to bovine serum albumin and calf thymus DNA. *Journal of Photochemistry and Photobiology B: Biology*.

[33] Salvi G., De Los Rios P., Vendruscolo M. (2005). Effective interactions between chaotropic agents and proteins. *Proteins: Structure, Function, and Genetics*.

[34] Melzak K. A., Sherwood C. S., Turner R. F. B., Haynes C. A. (1996). Driving forces for DNA adsorption to silica in perchlorate solutions. *Journal of Colloid and Interface Science*.

[35] Bhaganna P., Volkers R. J. M., Bell A. N. W. (2010). Hydrophobic substances induce water stress in microbial cells. *Microbial Biotechnology*.

